# “Communicate to vaccinate”: the development of a taxonomy of communication interventions to improve routine childhood vaccination

**DOI:** 10.1186/1472-698X-13-23

**Published:** 2013-05-11

**Authors:** Natalie Willis, Sophie Hill, Jessica Kaufman, Simon Lewin, John Kis-Rigo, Sara Bensaude De Castro Freire, Xavier Bosch-Capblanch, Claire Glenton, Vivian Lin, Priscilla Robinson, Charles S Wiysonge

**Affiliations:** 1 Centre for Health Communication and Participation, Australian Institute for Primary Care & Ageing, La Trobe University, Bundoora, VIC 3086, Australia; 2 Norwegian Knowledge Centre for the Health Services, PO Box 7004, St. Olavs plass, N-0130, Oslo, Norway; 3 Health Systems Research Unit, Medical Research Council of South Africa, Cape Town, South Africa; 4 International Union for Health Promotion and Education (IUHPE/UIPES), 42 Boulevard de la Liberation, 93203 Saint Denis Cedex, France; 5 Swiss Tropical and Public Health Institute, Socinstrasse 57, 4051, Basel, Switzerland; 6 School of Public Health, La Trobe University, Bundoora, VIC 3086, Australia; 7 School of Public Health, La Trobe University, 215 Franklin Street, Melbourne, VIC 3000, Australia; 8 Division of Medical Microbiology, Department of Clinical Laboratory Sciences, University of Cape Town, Anzio Road, Observatory 7925, South Africa; 9 Vaccines for Africa Initiative, Institute of Infectious Disease and Molecular Medicine, University of Cape Town, Anzio Road, Observatory 7925, South Africa

**Keywords:** Childhood, Vaccination, Immunisation, Communication, Low- and middle-income country, Intervention, Consumer, Taxonomy, Parents

## Abstract

**Background:**

Vaccination is a cost-effective public health measure and is central to the Millennium Development Goal of reducing child mortality. However, childhood vaccination coverage remains sub-optimal in many settings. While communication is a key feature of vaccination programmes, we are not aware of any comprehensive approach to organising the broad range of communication interventions that can be delivered to parents and communities to improve vaccination coverage. Developing a classification system (taxonomy) organised into conceptually similar categories will aid in: understanding the relationships between different types of communication interventions; facilitating conceptual mapping of these interventions; clarifying the key purposes and features of interventions to aid implementation and evaluation; and identifying areas where evidence is strong and where there are gaps. This paper reports on the development of the ‘Communicate to vaccinate’ taxonomy.

**Methods:**

The taxonomy was developed in two stages. Stage 1 included: 1) forming an advisory group; 2) searching for descriptions of interventions in trials (CENTRAL database) and general health literature (Medline); 3) developing a sampling strategy; 4) screening the search results; 5) developing a data extraction form; and 6) extracting intervention data. Stage 2 included: 1) grouping the interventions according to purpose; 2) holding deliberative forums in English and French with key vaccination stakeholders to gather feedback; 3) conducting a targeted search of grey literature to supplement the taxonomy; 4) finalising the taxonomy based on the input provided.

**Results:**

The taxonomy includes seven main categories of communication interventions: inform or educate, remind or recall, teach skills, provide support, facilitate decision making, enable communication and enhance community ownership. These categories are broken down into 43 intervention types across three target groups: parents or soon-to-be-parents; communities, community members or volunteers; and health care providers.

**Conclusions:**

Our taxonomy illuminates and organises this field and identifies the range of available communication interventions to increase routine childhood vaccination uptake. We have utilised a variety of data sources, capturing information from rigorous evaluations such as randomised trials as well as experiences and knowledge of practitioners and vaccination stakeholders. The taxonomy reflects current public health practice and can guide the future development of vaccination programmes.

## Background

Vaccination is one of the most important public health achievements of the 20th century [1]. It is a cost-effective public health measure and has led to the global eradication of smallpox and large reductions in poliomyelitis, measles, tetanus, rubella, diphtheria, *Haemophilus influenzae* type b (Hib) [1] and other conditions. However, over 22 million children are still not fully vaccinated [2], and there are significant inequities in vaccination coverage within and between countries [3]. Further, vaccination coverage rates in some countries are stagnating [4,5]. Efforts to improve vaccination coverage in low- and middle-income countries (LMICs) are central to meeting the Millennium Development Goal (MDG) of reducing child mortality [6].

### Conceptualising communication in health

Communication between and among health care providers and consumers has been highlighted as an emerging field of importance within the health care landscape [7,8]. Active participation of, and effective communication with, health care consumers has been demonstrated as a safe and efficient way to improve a broad range of health outcomes [9-12]. In the context of vaccination, communicating with parents and communities about the benefits of vaccination empowers them to carry out effective preventive health care, which in turn can increase vaccination uptake [13].

Historically, communication theorists have described the concept of communication as the linear journey a message takes from its source to its destination [14]. Since then, the concept of communication has evolved. The aims of communication are broad and so we use the term ‘communication’ as a short hand term to describe the range of ways in which we ‘seek patients, health consumers and family carers who are more knowledgeable and competent, able to express their views and beliefs, making choices alone or with health professionals, supported or supportive, minimising risks and harms, accessing high quality information and quality services, and participating in policy, research, governance and delivery’ [15] (p.14). The implication is that communication may refer to actions an individual takes; an exchange between two people, or interactions at the community level; and may encompass many media in addition to interaction between people. For example, the evolution of eHealth and mHealth, new forms of communication technology, have led to greater levels of interpersonal connectivity [16-18]. In this project, we define a communication intervention as a ‘purposeful, planned and formalised strategy associated with a diverse range of intentions or aims’ [19] (p.30). The concept of ‘purposeful, planned and formalised’ derives from the principles of evidence-based health care, where knowledge of an intervention’s effects on specific outcomes is a primary goal [15]. Further, recognition of the multidirectionality of communication promotes an understanding that meaningful communication associated with specific health purposes may originate as much from health consumers as it may from health care providers [20].

### The ‘Communicate to vaccinate’ project

**Figure 1:**
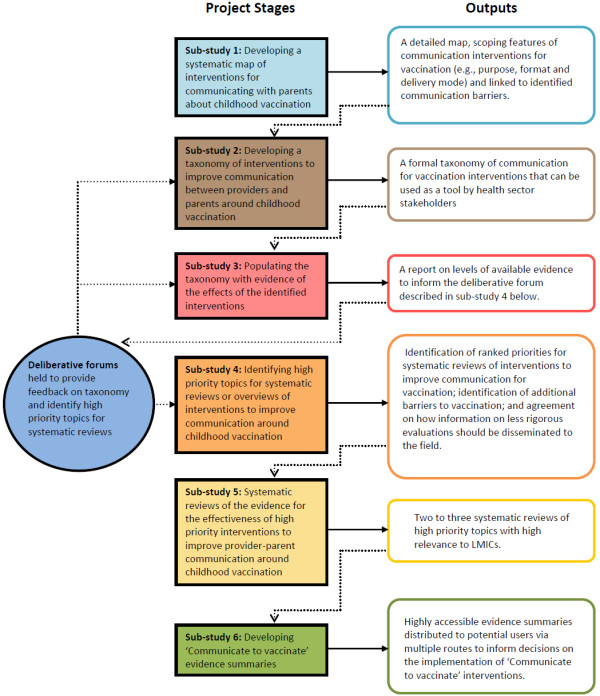
**Flowchart of the stages of the COMMVAC project.** This figure has been previously published [22]. We have the permission of all authors to reproduce this figure and acknowledge BioMed Central as the original publisher of this figure.

The ‘Communicate to vaccinate (COMMVAC)’ project [21] acknowledges the important role of communication in health and aims to clarify and build upon the available evidence of communication interventions to improve vaccination uptake in LMICs [22]. The project has six sub-studies, as outlined in Figure 1.

Despite the importance of communication to vaccine delivery and uptake we were unable to find any published comprehensive approaches to organising, and therefore understanding, the full range of communication interventions related to childhood vaccination. In particular, we were unable to identify any approaches which took into account the range of ways in which communication is facilitated [22]. We aimed to meet this need through the development of a taxonomy – a classification system organised into categories based on conceptual or practical similarities - of communication interventions. We believe that this taxonomy will help to understand the relationships between different types of interventions in the field. The taxonomy will also form a basis for future conceptual mapping of these interventions. This, in turn, will help to clarify their key purposes and features, assist with implementation and evaluation and identify areas where evidence is strong and where there are gaps [22-24].

We have seen a recent surge in the use of taxonomies to organise other areas of knowledge such as consumers’ medicines use [25], falls prevention strategies [26], barriers to the acceptance of office systems [27], health systems interventions [28] and behaviour change techniques [29].

The objective of this stage of the COMMVAC project was to develop a formal taxonomy of communication interventions for routine childhood vaccination, and this article outlines our methods.

## Methods

**Figure 2:**
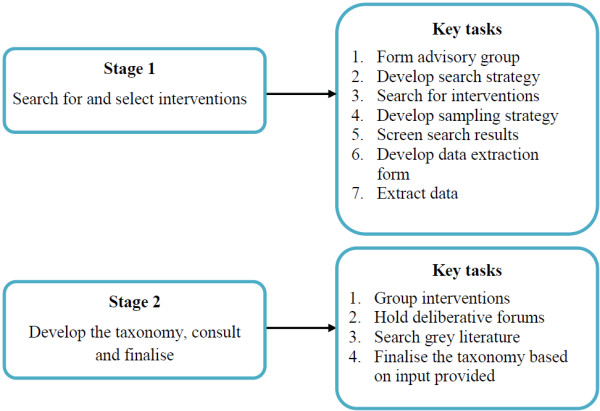
COMMVAC taxonomy methods.

Figure 2 summarises the methods used to develop the COMMVAC taxonomy. There were two main stages: Stage 1 focused on searching for and selecting interventions; Stage 2 involved developing the taxonomy, consulting on drafts of the work and finalising the taxonomy. Both stages included a number of key tasks, which are described in detail below.

### Stage 1: search for and select interventions

The objective of Stage 1 was to identify, classify and group the complete range of communication interventions focused on routine childhood vaccination, involving six key tasks.

#### Key task 1: form the advisory group

The COMMVAC project partners invited an international group of vaccination and communication experts to form an advisory group to provide comment and feedback on different aspects of the project. Representatives from the World Health Organization (WHO), the International Union for Health Promotion and Education (IUHPE), the Global Alliance for Vaccines and Immunisation (GAVI Alliance), the Cameroon Public Health Association and the Norwegian Knowledge Centre for the Health Services advised on the direction and implementation of the project and interpretation of findings. The advisory group provided specific advice on the organisation of the taxonomy and on the degree to which it reflected the experiences of those working in the vaccination and communication fields.

#### Key task 2: search for interventions

We obtained the interventions used to formulate the taxonomy from a variety of sources. We retrieved published trials and medical literature from two databases: The Cochrane Central Register of Controlled Trials (CENTRAL) and Medline. CENTRAL is a comprehensive databases of trials on the effects of health interventions and we therefore anticipated that it would yield a high proportion of relevant interventions. However, not all communication interventions have been evaluated in trials and new interventions are constantly being developed. We therefore also searched Medline without any study design filter so as to retrieve any kind of paper describing a communication intervention for vaccination.

We developed search strategies for Medline and CENTRAL (Additional files 1 and 2) based on Cochrane Collaboration principles [30]. The strategies included index terms and text word combinations that were associated with vaccination, based on an approach developed in a related project [31], and combinations associated with communication. Communication terms were derived from the Cochrane Consumers and Communication Review Group’s (CC&CRG) scope and included all terms associated with people’s interactions with the health system or about their health, such as communication, health education, information provision, decision making and media communication [32].

In addition to these sources, we identified interventions based on the experiential and practice knowledge of key experts in the field of vaccination and communication. We gathered this information through a series of deliberative forums held in June and July 2011 and through the advisory group. Lastly, we supplemented the systematic search for studies indexed in CENTRAL and Medline with purposeful hand searching of the grey literature available on the internet and through major health agencies. These last two steps were conducted in Stage 2 (see below).

#### Key task 3: develop a sampling strategy

We developed a sampling strategy because the Medline search produced greater than 10 000 records. We performed a sample size calculation in order to reduce the high yield of articles while maintaining a sample size capable of answering our research question. Our goal was to capture the complete range of unique interventions described by these records. We screened a small subset of 100 Medline articles and found one unique intervention type in every fifty articles (as well as many articles that described intervention types already identified), i.e., 2% of the subset sample constituted unique vaccination communication interventions. To account for any variation in the subset, we rounded up and estimated that we would find a new intervention in 5% of records. We used a sample size calculator to determine with 95% confidence and 90% precision that we would need to screen 1002 records from the Medline search output in order to capture all relevant new interventions. We then organised the Medline search results chronologically according to publication date and used a random number generator programme to choose a number between 1 and 19. This number was eight. From the eighth article in the list, we selected every 19th article. This resulted in a sample of 1002 articles from Medline.

#### Key task 4: screen the search results

We developed selection criteria and applied them to articles identified from CENTRAL and Medline, to interventions identified from the grey literature, as well as those obtained from key stakeholders participating in the deliberative forums. The selection criteria were that the article:

1. Identified or mentioned a communication intervention, consistent with the COMMVAC definition of communication [19,22]

2. Described a target population of children up to six years of age. Articles in which the target population age was not described but could potentially include children up to six years old were included

3. Reported on childhood vaccination and included communication with parents or communities. Interventions directed to health care providers were only included if they directly impacted on interactions with consumers

4. Focused on routine childhood vaccines only, defined by WHO’s recommendations for all children [33]

5. Was written in English. Non-English records were not included as the project did not have the resources to translate full text papers published in other languages.

Two researchers (NW and JK) independently screened titles and abstracts for eligibility, retrieving full text records if insufficient information was available. Discrepancies were resolved through discussion or by involving a third researcher (SH).

#### Key task 5: develop a data extraction form

We developed a data extraction form (see Additional file 3), building on the data extraction form recommended for authors of systematic reviews for the CC&CRG [34]. We piloted the form within the project team to ensure ease of use and clarity. The final form contained the following data extraction cells:

1. Article features

••Type of study

2. Population features

••Population group/s

••Setting/s of vaccination services (i.e. country and region, urban or rural)

••Details of vaccines and coverage

••Age of infants or children

3. Communication intervention features

••Intervention purpose

••Direction of communication (i.e. the target of the intervention)

••Parties involved

••Content of communication

••Format and delivery method

••Deliverer (i.e. service type or personnel)

••Training required

••Setting/s

••Frequency or timing of communication

••Cost

4. Outcome features

••Main outcomes measured (where applicable).

#### Key task 6: extract the data

Data on the communication interventions identified were extracted by two researchers (NW and JK) and entered into the data extraction form in Microsoft Word.

For trials, we extracted whatever information was available about the communication intervention being evaluated, along with outcomes features. Much of this information was intended to be utilised in later stages of the project, as the focus of the taxonomy was primarily on intervention type and purpose. For other types of articles, we extracted any information reported on all of the communication interventions described in the article, even if some interventions were only mentioned briefly. The rationale behind this was to ensure that interventions that have not necessarily been evaluated but which are being implemented in the field were captured in the taxonomy. For example, interventions involving the community in vaccination programmes [35], or the use of celebrities in promoting an important vaccination message [36], can be integral features of vaccination strategies despite being under-represented in trial research.

### Stage 2: develop the taxonomy, consult and finalise

The objective of Stage 2 was to develop and finalise the taxonomy through extensive consultation with key communication and vaccination stakeholders. We conducted a thorough search of the grey literature in this stage to help achieve this objective, along with the other key tasks described below.

#### Key task 1: group the interventions

Once all the available data were extracted, we compiled a complete list of interventions in Microsoft Excel in which we described each intervention using simple, non-technical terms. This ‘raw’ interventions list became the launching pad for our taxonomy. By discussing common themes and features in this list, NW, JK and SH grouped interventions conceptually according to their purpose. We then developed a coding system to organise and manage these data. Once the interventions were grouped, the major overarching categories in the taxonomy were formed. In establishing the purpose of interventions we were guided by the language used by the authors of the articles, rather than imposing our own interpretations. The overarching primary categories were then further divided to provide greater clarity and definition between the different intervention types.

In developing the COMMVAC taxonomy, we built on taxonomies already developed by the project partners, including: (1) a comprehensive taxonomy for all interventions for communication in health [37]; and (2) a taxonomy of interventions directed to consumers for evidence-based prescribing [25]. We also referred to Abraham and Michie’s taxonomy of behaviour change techniques [29] and a global evidence mapping initiative led by Bragge and colleagues [23].

This initial version of the taxonomy (Table 1) comprised seven categories and 34 intervention types. The original categories were: inform or educate; remind or recall; teach skills; support; minimise risks and harms; increase access to or likelihood of contact with health care/vaccination services and; involve the community in planning, programme delivery, research, advocacy or governance. This version of the taxonomy was presented to a series of deliberative forums and to the advisory group for comment. It included a range of examples for each intervention type to assist participants in understanding what might be included in each category.

**Table 1:** Initial version of the COMMVAC interventions taxonomy

**Purpose**	**Intervention types**
**Inform or Educate**	
Strategies to enable consumers to understand the meaning and relevance of vaccination to their health and the health of their family or community. Interventions may be delivered in many formats and by many methods, including face to face interaction, mail, phone, device or tool, audio visual presentation or performance, printed materials, websites, multi-media campaigns, or community events. Interventions to inform or educate may be directed at individuals, groups, or communities and, communities, or providers and may include information about vaccine-preventable diseases; risks and benefits of vaccines; where, how, and when to access vaccine services; and who should receive vaccination.	• Face to face interactions
	• Postcards, letters or email
	• Phone calls or SMS
	• Device or tool
	• Audio visual/performance
	• Printed material
	• Web-based
	• Media campaign
	• Community event
	• General
**Remind or Recall**	
Strategies to remind consumers or providers of required, recommended, or scheduled vaccination services and to recall those who are overdue for vaccination. Interventions may be delivered in face to face interactions at clinics or in a person’s home, by mail, phone, or with a device or tool. They may include personalised information related to a specific upcoming or missed appointment, or may be more focused on promoting general awareness of available vaccines. Contact may be made once or multiple times.	
	• Face to face interactions
	• Postcards, letters or email
	• Phone calls or SMS
	• Device or tool
	• General
**Teach Skills**	
Strategies to provide individuals with the ability to operationalise knowledge through the adoption of practicable skills. Skills may be taught to consumers or those engaged in the delivery of health services. People may be taught general parenting skills, how to share information effectively amongst their peers, or how to deliver information or education to others in both formal and informal settings.	• Parenting skills programmes
	• Peer to peer information sharing
	• Training in how to communicate/provide education to others
	• General
**Support**	
Strategies to provide assistance or advice for consumers outside the traditional consultation environment. Interventions include face to face interactions which may take place at an individual’s home or in a group session, telephone support calls or access to a telephone helpline, and referrals to put people in touch with community or other healthcare services.	
	• Face to face interactions
	• Phone contact
	• Web-based
**Minimise risks or harms**	
Strategies to help consumers recognise, record or respond to personal risks associated with vaccination, such as adverse events.	• Parent recording or reporting of adverse events
**Increase access to or likelihood of contact with healthcare/vaccination services**	
Strategies to assist individuals in overcoming challenges to reaching and utilising health services. Interventions may address barriers to access including but not limited to time, transportation, money, or language. Interventions may include greater availability of care through mobile clinics or extended clinic hours; providing vaccinations at unrelated healthcare visits; outreach escorts to help bring children to clinics or assist in making appointments; incentives or disincentives; multi-lingual interpreters; or the provision of free or reduced-cost vaccines.	
	• Mobile clinic
	• Opportunistic vaccination
	• More convenient care
	• Transportation assistance
	• Incentives or disincentives
	• Interpreters
	• Free vaccines
**To involve the community in planning, programme delivery, research, advocacy or governance**	
Strategies to engage the members of a community in the execution or implementation of health and vaccination services; or to generate awareness and understanding and strengthen relationships and communication within a community in relation to vaccine delivery and education. Interventions may be simple, such as holding community focus groups for priority-setting, or complex, such as building relationships between different sectors and organisations within a community.	
	• Community coalition
	• Programme delivery
	• Community input
	• Partnership building

#### Key task 2: hold deliberative forums

Consulting vaccination and communication experts to gather their unique practice perspectives was an important step in refining the taxonomy, and one that has been commonly adopted in the development of other taxonomies [22,26,38-40]. We presented the initial version of the taxonomy to a range of international stakeholders at deliberative forums. Stakeholders included representatives of governmental and non-governmental agencies, consumer groups, as well as experts in the field of vaccination and communication.

The deliberative forums included two face to face consultations convened in New York and Ottawa (see Additional file 4) and one online forum. Materials were translated into French for the Ottawa and online forums. Project partners based in Melbourne and Cape Town moderated the online forum, posting discussion questions related to the taxonomy and collating and translating the views of forum participants between French and English where necessary.

Participants were asked to comment on whether the categories and their organisation were logical and whether any interventions were missing. These consultations were also pivotal in the identification and formulation of topics for systematic reviews of the effects of communication interventions for vaccination, currently being completed as part of COMMVAC sub-study 4 (see Figure 1) [16,41].

#### Key task 3: search the grey literature

We conducted a focussed search of the grey literature in order to complement database searching. Websites were selected following extensive discussions within the project team, with input from the advisory group. We searched the websites of the Communication Initiative Network, World Health Organization (WHO), United Nation Children’s Funds (UNICEF), Global Alliance for Vaccines and Immunization (GAVI Alliance), Immunization Basics, Programme for Appropriate Technologies in Health (PATH), Centre for Global Health Communication and Marketing, US Centers for Disease Control and Prevention (CDC), and the Maternal and Child Health Integrated Programme (MCHIP). New or unique interventions described by these sources were added to existing taxonomy categories where appropriate, or used to refine new taxonomy categories.

#### Key task 4: finalise the taxonomy based on the input provided

A core group of the project team consisting of NW, SH and JK revised the initial taxonomy based on feedback from project partners, the advisory group and deliberative forum participants.

Apart from identifying interventions not already captured in the taxonomy, the advisory group and deliberative forum participants suggested a variety of different ways to organise the initial version of the taxonomy, as well as different features to be incorporated. Two overarching messages emerged from the feedback process and greatly influenced the taxonomy.

The first message was that the taxonomy needed to more clearly identify the person or people (e.g. consumers, health care providers) to whom the intervention is delivered. In response to this feedback, we changed the structure of the taxonomy by adding columns identifying the target of interventions in order to better differentiate between parents or soon to be parents, communities and health care providers. We did not feel that identifying the deliverer would be helpful or possible, however, as health care messages can be multidirectional and may involve a range of different people depending on the health care context [20].

**Figure 3:**
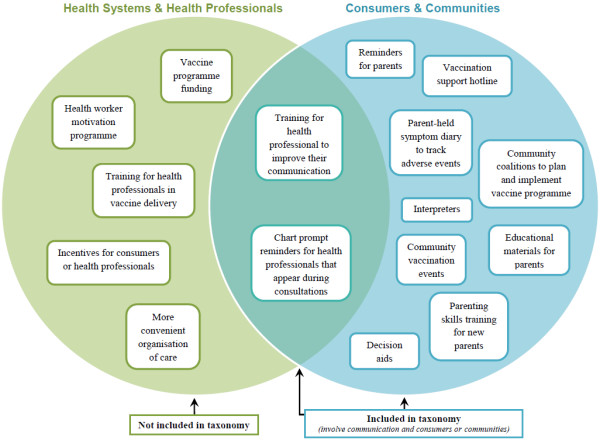
Diagram illustrating the scope of interventions included in the COMMVAC taxonomy.

The second recommendation was to make clearer the taxonomy’s scope and parameters. As the focus was on communication interventions, the feedback raised questions about the appropriateness of including interventions such as the delivery of free vaccines or incentives for general practitioners. We agreed that the taxonomy would only incorporate interventions that either impacted on the provider-consumer interaction or involved communication with, and participation of, parents, caregivers and community members. However, drawing this line in practice was challenging, particularly in terms of the recurring issue of distinguishing between supply-side (funding of vaccines) and demand-side (education of parents) interventions. We produced a Venn diagram (Figure 3) to convey visually which interventions we included and excluded.

As the scope of the taxonomy became clearer through these consultations and diagrammatic representation, we removed various categories and intervention types that did not involve communication with parents and communities. For example, we removed the “Increase access to or likelihood of contact with health care/vaccination services” category, along with intervention types contained in this category, such as “Opportunistic vaccination” and “Free vaccines.”

In addition to these major changes, we made several smaller alterations based on the input gathered from forum participants and advisory group members. To better reflect the experiences and goals described by people working in vaccine programmes in LMICs, we changed the language surrounding the community involvement category from “To involve the community in planning, programme delivery, research, advocacy or governance” to “Enhance community ownership”. We added the concept of “building trust” into the definition of this category and included “local opinion leaders” as an intervention type after forum participants highlighted the importance of advocacy toward community and religious leaders. We also added “addressing misinformation” to the definition of “To inform or educate”

We then held further deliberations within the project team to agree on the final taxonomy. Through this iterative process, we have attempted to ensure that: appropriate definitions and language are used and category descriptions are clear and relevant; the taxonomy represents global activity as well as LMIC experiences; interventions for which evaluations were not found are identified; and interventions currently being used in practice are highlighted.

In the following section we describe the search results and final taxonomy in detail.

## Results

We conducted searches in CENTRAL (October 5th 2010 – 556 records) and Medline (December 6th 2010 – 27 061 records).

**Figure 4:**
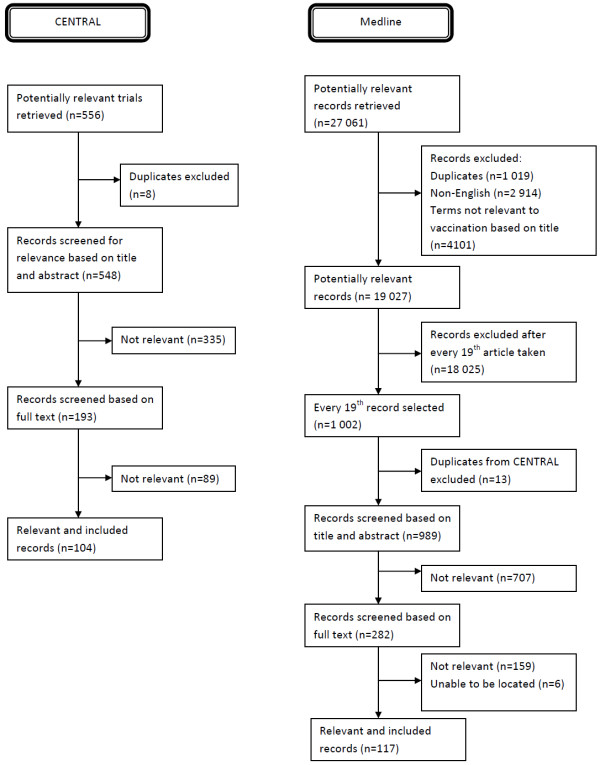
Exclusion flowchart for records contributing to the development of the COMMVAC taxonomy.

After excluding duplicates, non-English language and non-relevant articles, we were left with 19 027 potentially eligible Medline records (Figure 4). As the number of articles retrieved from Medline was above the threshold that we set of 10 000 articles, we applied the sampling approach described above prior to screening. This resulted in 1002 records. After screening, 117 eligible records were identified from Medline.

After excluding duplicates 548 CENTRAL records remained and were screened, resulting in 104 eligible records. Full text papers were then obtained for the total 221 records, which provided information on 43 unique intervention types covering 471descriptions of interventions.

We will now describe the final ‘Communicate to vaccinate’ taxonomy.

### The ‘Communicate to vaccinate’ taxonomy

**Figure 5:**
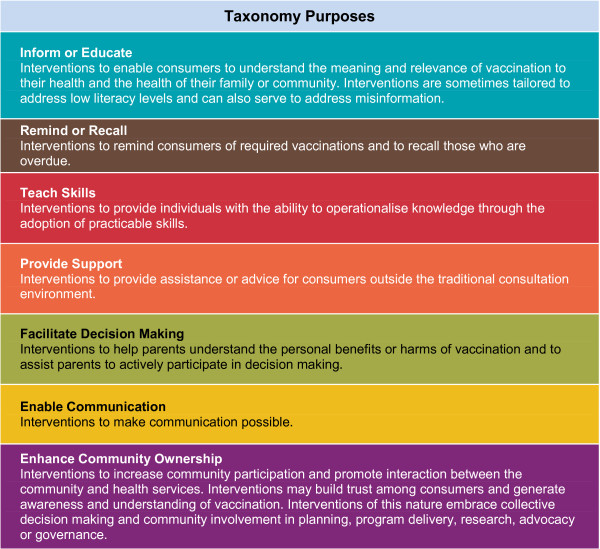
COMMVAC taxonomy purposes and definitions.

**Figure 6:**
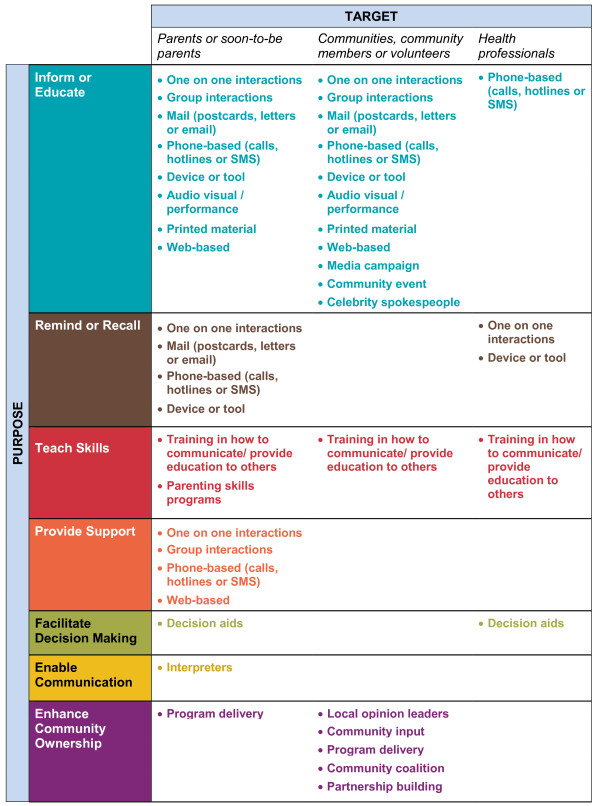
COMMVAC taxonomy of interventions to improve communication about childhood vaccination.

The purposes of communication interventions serve as the central classification method in our final taxonomy (Figure 5). We chose to organise the ‘Communicate to vaccinate’ taxonomy in this way as the intended aim, or purpose, of an action is an integral feature of any communication intervention [19] and is ultimately the main determinant of what one does in practice. The taxonomy includes definitions of the seven main communication purposes: inform or educate; remind or recall; teach skills; provide support; facilitate decision making; enable communication; and enhance community ownership. Within these categories, the interventions are broken down into 43 types and are also categorised according to their main target: parents or soon to be parents; communities, community members or volunteers; and health care providers (Figure 6).

We summarise below the main overarching ‘intervention purpose’ categories in the taxonomy and provide examples of the interventions included in these categories. An expanded version of the taxonomy including category definitions, intervention types and examples is available on the COMMVAC project website [21].

#### Inform or educate

Communication interventions in this category are tailored towards empowering parents and communities to understand the benefit and relevance of vaccination to their health and the health of their family and community. Informing and educating parents and communities has several underlying aims. These include addressing misinformation and poor knowledge regarding the practical and logistical factors associated with vaccination. This is achieved in a wide range of ways. For example, nurses or vaccine providers can communicate directly with children about vaccines through methods such as puppet shows [42]. Letters/emails, pamphlets, telephone calls/text messages and face to face interactions are typically targeted towards parents or soon to be parents [43-46], whereas television documentaries, immunisation carnivals and media campaigns can be used to disseminate information to entire communities [36,42,47].

#### Remind or recall

Interventions in this category prompt parents of upcoming or overdue vaccinations through a variety of delivery mechanisms and evolving forms of technology. Letters/emails, telephone calls/text messages, face to face interactions or tools (e.g. fridge magnets) are all mechanisms by which parents can be reminded to have their child vaccinated [48-50]. Health care providers can also be reminded of due vaccinations at specific health appointments. These reminders usually take the form of chart prompts or alerts at the point of care, thus influencing the parent-provider interaction [51]. Provider reminders that do not occur at the point of care are not included in this taxonomy as they do not directly affect interactions between providers and parents.

#### Teach skills

Interventions that aim to teach parents, communities and health care providers the skills required to operationalise knowledge are an important component of communication. Parenting skills programmes are located within this category. Parents are taught various skills related to child health such as breast feeding, oral rehydration and general parenting skills. Early childhood vaccination is often an integral component of these programmes, which not only include an educational element but also guidance for parents on how to ensure their child is vaccinated appropriately. Another important feature of this category is interventions aimed at training parents, communities and health care providers in how to communicate or provide education to others. This can include suggestions for how a mother can communicate to her child to reduce stress at vaccination appointments [52] or the training of local health workers in how to educate others [53].

#### Provide support

Communication interventions typically described in the literature as providing support to parents are important aspects of vaccination uptake. Their purpose is to provide assistance or advice to parents outside of the traditional consultation environment about vaccination. Support and information exchange between parents via an online chat forum is an example [54].

#### Facilitate decision making

Interventions that are specifically described as decision aids for parents were classified separately as these tools have a wider purpose than simply educating or informing a parent about vaccination. Decision aids are evidence-based tools that prepare consumers to make decisions when confronted with multiple health care [12].

#### Enable communication

This is a narrowly defined category which includes interventions that aim to make communication between parents and health care providers possible, specifically in the face of a practical obstacle such as disability or a language difference. For example, the use of interpreters to translate important vaccination messages is a unique intervention type that is more purposeful than simply translating information [55].

#### Enhance community ownership

Interventions included in this category aim to enhance interactions between communities and health services as a way of achieving better awareness and fostering a sense of community ownership of vaccination. Community participation in all aspects of vaccination programme delivery, planning, research or governance may contribute to building trust among consumers. Community involvement and grassroots support for vaccination programmes may also influence the uptake of vaccination on a community-wide scale. Interventions in this category include: community coalitions charged with overseeing the implementation of vaccination programmes; enlisting local opinion leaders such as village chiefs to serve as vaccination ‘champions’; recruiting village members to assist with vaccination programme delivery; or community input into the design of vaccination strategies [47,56-58].

## Discussion

Communication is an important component of vaccination programmes, alongside service delivery, logistics, vaccine supply and surveillance and may also provide opportunities for other health promotion messages and activities. Developing a classification system based on conceptual similarities among communication interventions will help to: clarify the key purposes and features of interventions; introduce a common language of communication interventions in the vaccination field; and assist with the conceptualisation of communication as an intervention in its own right.

### Limitations and areas where further work is needed

We developed the taxonomy with extensive input from a range of knowledgeable individuals, but some of their suggestions fell outside the scope of the taxonomy and were therefore not incorporated in the final structure. We discuss below these areas where further work may be needed.

#### Reframing the taxonomy according to known vaccination barriers

Some advisory group members and forum participants suggested that it may be useful to reframe the taxonomy according to known vaccination barriers, making the taxonomy a ‘menu’ of solutions to common barriers. Garner and colleagues [59] conducted a similar mapping exercise to link known barriers to adherence to tuberculosis treatment to relevant interventions, so as to “help policy makers and providers think through the barriers and determine how best to address them” (p.404). We did not attempt this here for several reasons: first, it would have required a separate extensive review of the substantial ‘barriers’ literature, which was not be feasible given project resources. Second, when extracting data to develop the taxonomy it quickly became apparent that the available literature on interventions generally omits information related to barriers to vaccination uptake. Third, assumptions often need to be made to link known barriers to certain interventions. For example, when demonstrating the link between a barrier and an intervention, Garner and colleagues [59] state that: “staff training *probably* tackles health-system barriers by improving the quality of health care” (p. 404. Emphasis added), and that their own judgement was required to link some barriers to interventions. This, we felt, would make barrier mapping a subjective task unless substantive work could be done to explore the extent to which interventions address specific barriers. Last, a major philosophical concern with organising interventions around the central theme of barriers is that it assumes that all interventions are designed or intended to overcome opposition, resistance or hindrances to vaccination. However, information interventions, for instance, may primarily be used to inform parents - a purpose shared by other early parenting interventions – who can then make informed choices regarding vaccination uptake. Such interventions may focus on vaccination as a public good rather than on attempting to overcome specific barriers to vaccination uptake. We therefore felt that a barrier analysis may be a useful secondary analysis but not as a primary organising framework.

#### Clarifying who delivers the interventions

A forum participant requested that the taxonomy incorporate “who does what to whom”. This would involve describing who delivers the intervention, what the intervention is and who the intervention is directed to. However, increasingly interventions are being delivered by a mix of health professionals and lay health workers [60] or using communication mediums such as the internet and mobile phones. This meant that this information did not aid the central purpose of classification, although we extracted data where possible on these items and this information could aid a detailed map of specific intervention types.

#### Outlining how interventions change social norms

One participant suggested the inclusion of “Changing social norms” as an additional taxonomy category. However, one could argue that changing a social norm applies to most interventions to increase vaccination coverage. For instance, vaccination can be viewed as ineffective or dangerous - a social norm or belief shared by some in the community. An intervention to educate parents or communities about the safety and effectiveness of vaccination is intended in part to change this social norm. We therefore did not consider it useful to include “changing social norms” as a category within the taxonomy, but further work is needed to explore how these different groups of interventions change beliefs and behaviours [29,61].

#### Specifying the contextual elements that contribute to the effectiveness of interventions

A member of the advisory group proposed that the taxonomy specify the preconditions, or contextual elements, that contribute to the effectiveness of communication interventions for vaccination. These include institutional systems; cultural, political and economic environments; as well as the presence of supporting factors, such as legislation. Unfortunately, these preconditions are rarely described in the literature and their inclusion was therefore not feasible. Further primary research in this area is needed.

#### Examining the gender dimensions of all studies

Lastly, it was suggested that the taxonomy incorporate the gender dimensions of all included interventions. While an analysis of gender roles and responsibilities would be interesting, it was not possible to include this in the taxonomy for similar reasons to those outlined for contextual elements.

#### Other limitations

There are a number of important elements of communication interventions that this taxonomy is not intended to describe or categorise, including the content of the communication; how the communication happens, for example, the nature of the interpersonal communication involved, which may include empathic listening; the characteristics of the target population; the outcome measures used; the setting, timing or frequency of the intervention; or the level of health system involvement. These intervention features are being further explored in the evidence map phase of this project (sub-study 3 - see Figure 1), to the extent that this data is available.

Another possible limitation of the taxonomy is that it does not include a category for multifaceted interventions – rather, these were separated into their individual components for classification. For example, we separated interventions involving both reminders and education into two interventions because the purpose of this taxonomy is to provide a system of discrete categories into which each kind of communication intervention can be classified, so as to describe and clarify the purposes and features of each. Of course, evaluations of such multifaceted interventions may need to consider both the effectiveness of the package as a whole and the contributions of the key components.

Finally, resource constraints limited our search to articles published in English only. This means that we could not address the varied sociocultural approaches to communication around the world, and further research on this would be useful. It is also possible that we missed interventions unique to non English-speaking country settings. Acknowledging our resource constraints, we attempted to address this limitation in part by including participants from Francophone African countries in one of the deliberative forums and by making the online forum accessible to French speakers, with materials and discussion summaries translated into French. This expanded the scope of the project to include input from representatives in several additional African countries where vaccination is a critical issue.

### Implications for research and practice

The ‘Communicate to vaccinate’ taxonomy, which incorporates the principles of evidence-informed public health and is guided by current practice, has a number of implications for research and practice. Firstly, it will assist both researchers and practitioners in identifying the key purposes of communication interventions. This new way of organising interventions will help people to design communication initiatives based on the goal or purpose of the communication, cutting across the myriad different intervention formats and ever-changing forms of communication technology. Secondly, it may contribute to improving and standardising the description of communication interventions in the vaccination field, thereby improving the information available to vaccination programme managers and to researchers. Thirdly, as evidence of effectiveness is mapped against the interventions in this taxonomy, the product will assist in selecting for implementation those interventions shown to be effective, as well as in identifying evidence gaps. Fourth, and following from this, researchers will be able to use this taxonomy to facilitate evidence mapping, a relatively new method of identifying where evidence is located and where future research should be conducted to close evidence gaps [23]. As undertaking new primary research in the absence of a comprehensive synthesis of existing knowledge is wasteful [62], evidence mapping based on a comprehensive taxonomy of interventions can aid in the prioritisation of future research. In addition, the process of developing the taxonomy has identified a number of research questions (e.g. mapping theoretical perspectives of interventions), as discussed in more detail above.

## Conclusions

There is a substantial body of knowledge on communication in the field of childhood vaccination, but to date this has been difficult to navigate. To address this, we have developed a taxonomy of communication interventions for vaccination, which outlines seven main intervention categories: inform or educate, remind or recall, teach skills, provide support, facilitate decision making, enable communication and enhance community ownership. This taxonomy both illuminates and organises this area of work and identifies the range of communication interventions that programme managers might utilise to increase routine childhood vaccination uptake. In developing the taxonomy, we have utilised a variety of data sources, capturing not only high quality trial research but also the experiences and knowledge of practitioners and key vaccination stakeholders. As a result, we believe that the taxonomy reflects the complete range of ‘Communicate to vaccinate’ interventions currently in practice globally and will be an important guide for the future development of vaccination programmes.

## Abbreviations

CC&CRG: Cochrane Consumers and Communication Review Group; CENTRAL: The Cochrane Central Register of Controlled Trials; COMMVAC: Communicate to vaccinate; EPOC: Effective Practice and Organisation of Care Review Group; GAVI: Global Alliance for Vaccines and Immunization; GLOBVAC: Global Health and Vaccination Research; IUHPE: International Union for Health Promotion and Education; LMICs: Low- and middle-income countries; MDG: Millennium Development Goal; PATH: Programme for Appropriate Technologies in Health; MCHIP: Maternal and Child Health Integrated Programme.

## Competing interests

The authors declare that they have no competing interests.

## Authors’ contributions

SL and SH led the conceptualisation and design of the project, prepared the original project proposal and obtained funding. CSW, PR, VL and XBC contributed to the original proposal. JKR conducted the initial database search. NW and JK screened all articles and extracted the data. NW, JK and SH developed the initial version of the taxonomy, with contributions from all authors. JK, CW, VL and SBFC facilitated the deliberative forums. JK, NW, SL and CSW participated in the design and implementation of the grey literature search. NW developed this manuscript, with input from SH, JK, SL, VL, PR, CSW and XBC. All authors provided input into various aspects of the project, provided ongoing feedback and approved the final version of the manuscript.

## Pre-publication history

The pre-publication history for this paper can be accessed here:

http://www.biomedcentral.com/1472-698X/13/23/prepub

## Supplementary Material

Additional file 1 COMMVAC Medline search strategy. Click here for file

Additional file 2 COMMVAC CENTRAL search strategy. Click here for file

Additional file 3 COMMVAC data extraction form. Click here for file

Additional file 4 COMMVAC deliberative forum reports. Click here for file
