# Correction: “Communicate to vaccinate”: the development of a taxonomy of communication interventions to improve routine childhood vaccination

**DOI:** 10.1186/1472-698X-13-37

**Published:** 2013-09-18

**Authors:** Natalie Willis, Sophie Hill, Jessica Kaufman, Simon Lewin, John Kis-Rigo, Sara Bensaude De Castro Freire, Xavier Bosch-Capblanch, Claire Glenton, Vivian Lin, Priscilla Robinson, Charles S Wiysonge

**Affiliations:** 1 Centre for Health Communication and Participation, Australian Institute for Primary Care & Ageing, La Trobe University, Bundoora, VIC 3086, Australia; 2 Norwegian Knowledge Centre for the Health Services, PO Box 7004, St. Olavs plass, N-0130, Oslo, Norway; 3 Health Systems Research Unit, Medical Research Council of South Africa, Cape Town, South Africa; 4 International Union for Health Promotion and Education (IUHPE/UIPES), 42 Boulevard de la Liberation, 93203, Saint Denis Cedex, France; 5 Swiss Tropical and Public Health Institute, Socinstrasse 57, 4051, Basel, Switzerland; 6 School of Public Health, La Trobe University, Bundoora, VIC 3086, Australia; 7 School of Public Health, La Trobe University, 215 Franklin Street, Melbourne, VIC 3000, Australia; 8 Department of Clinical Laboratory Sciences, Division of Medical Microbiology, University of Cape Town, Anzio Road, Observatory, Cape Town 7925, South Africa; 9 Vaccines for Africa Initiative, Institute of Infectious Disease and Molecular Medicine, University of Cape Town, Anzio Road, Observatory, Cape Town 7925, South Africa

## Correction

After publication of the original article [1] it came to the publisher’s attention that the article was incorrectly published as ‘Correspondence’ instead of the correct article type; ‘Research’. We apologise for any inconvenience this has caused.

## Pre-publication history

The pre-publication history for this paper can be accessed here:

http://www.biomedcentral.com/1472-698X/13/37/prepub
