# Supplementary material for: “Communicate to vaccinate”: the development of a taxonomy of communication interventions to improve routine childhood vaccination
Source: BMC Int Health Hum Rights. 2013 May 11;13:23. doi: 10.1186/1472-698X-13-23 (PMC3655915; doi:10.1186/1472-698X-13-23)
Supplement: Additional file 1 — COMMVAC Medline search strategy. [file 1472-698X-13-23-S1.doc]

Commvac

1. exp immunization/

2. exp vaccines/

3. (immuniz* or immunis* or immunotherap* or vaccin* or inoculat*).mp.

4. or/1-3

5. exp communication/

6. ((health or patient or mediated or facilitated or augmentative or alternative or total or simultaneous or manual or mass or face-to-face or oral or cultural or risk or intervention* or interaction* or program* or skill* or aid* or tool* or board* or device* or system* or barrier*) adj1 communication).mp.

7. (communicat* or messag* or verbal* or nonverbal* or written or writing or reading or language or speech or speak* or spoken or talk* or conversation or voice or visual-perception or feedback or listen* or negotiat* or notify* or notification or remind* or narrat* or music* or humor or humorous or adverti* or persua* or interpreting or interpreters or interpret*-service or translat* service* or translating).hw,ti.

8. (readability or intelligibility or credibility).mp.

9. (disclos* or trust* or truth* or deceiv* or deception or misinform*).hw,ti.

10. exp interpersonal relations/

11. hospital patient relations/

12. community institutional relations/

13. ((professional or physician or doctor or clinician or nurse or provider) adj1 (patient or client or family)).tw.

14. ((health or patient or client) adj (education or knowledge or promotion)).mp.

15. exp health promotion/

16. ((education* or teaching or learning or instruction* or training or skills or online or web* or internet or video* or multimedia or multi-media) adj1 (intervention* or session* or course* or program* or material* or package* or module* or demonstration or method* or process*)).mp.

17. (((medical or continuing or residency or distance) adj2 education) or internship or inservice or in-service or staff development or professional development or mentor* or lifelong learning).mp.

18. (self adj (teaching or education or instruction)).mp.

19. ((media adj3 campaign*) or (promotion adj1 program*) or (community based adj3 intervention*) or (awareness adj3 (rais* or increas*))).tw.

20. marketing.mp.

21. ((family or office or work* or school or faith or church) adj based).tw.

22. (educational status or literacy).mp.

23. ((improv* or increas* or enhanc* or patient) adj3 (understanding or comprehension)).tw.

24. (information* adj (service* or center* or system* or dissemination or seeking or retrieval or transfer* or campaign* or provision or aid or material* or sheet* or pack*)).mp.

25. ((patient or client or health or medical or drug or written or print* or visual* or provid* or present*) adj2 inform*).mp.

26. (((inform* or message* or communicat* or effect* or gain or positive or negative) adj2 fram*) or ((verbal or oral or written or text or data or numerical or statistical or visual or graphic* or pictorial or audio* or video* or multimedia or multi-media or narrative) adj (format* or presentation or display*))).mp.

27. (counsel* or ((social or carer* or caregiver* or care giver* or patient*) adj1 support*) or psychosocial or ((social or pastoral or spiritual) adj care) or religion or chaplaincy or behavior modification).mp.

28. (counsel*ing session* or ((support or peer or self-help or self-care) adj2 (intervention* or group* or program*))).mp.

29. ((social or community) adj2 network*).mp.

30. (self-care or self-management).mp.

31. (motivat* or incentive* or goal*).mp.

32. exp communications media/

33. ((mass or communication* or electronic or digital or multi or print* or social or new) adj media).tw.

34. ((print* adj (material* or based)) or paper-based or written material* or (paper adj1 pen*) or publication* or newsletter* or brochure* or booklet* or pamphlet* or leaflet* or flyer* or handout* or poster* or illustrat* or picture* or pictogram*).mp.

35. (radio or television or audiovisual or video* or tape recording* or cassette* or cd-rom* or dvd* or motion picture* or multimedia or hypermedia or telephon* or phone or phones or sms or short message* or text message* or i-pod* or ipod* or mp3 player* or hotline* or answering service* or internet or web* or online or on-line or blog* or telemedicine or telehealth or telecare or (virtual adj (reality or world or environment*))).mp.

36. ((electronic or e-) adj (mail or prescri* or health or game*)).mp.

37. exp computer systems/

38. software/

39. (computer* adj1 (system* or network* or program* or terminal* or interfac* or interact* or handheld or intervention* or therapy or graphic* or simulation* or searching or mediated or based or tailored or communication or assisted instruction)).mp.

40. (touch screen or digital assistant* or pda or blackberry or mobile-device* or laptop* or notebook computer*).mp.

41. (((automat* or interactive*) adj3 (telephon* or phone or phones or voice or hotline* or hot line*)) or ((voice or speech) adj (response or recognition or messag* or system* or technolog*))).mp.

42. (cultural* adj (competen* or sensitiv* or appropriate)).mp.

43. ((cultural* or linguistic* or language) adj3 (service* or care or intervention* or message*)).mp.

44. (participation or advocacy or consumer* or empower*).mp.

45. exp decision making/

46. (decision adj (making or support or aid*)).mp.

47. exp informed consent/

48. (informed adj (consent or choice* or decision*)).tw.

49. ((patient or person or family or client) adj (cent*red or focus*ed or oriented)).mp.

50. (therapeutic adj (relation* or alliance*)).mp.

51. or/5-50

52. 4 and 51

53. exp animals/ not humans.sh.

54. 52 not 53
