# Supplementary material for: “Communicate to vaccinate”: the development of a taxonomy of communication interventions to improve routine childhood vaccination
Source: BMC Int Health Hum Rights. 2013 May 11;13:23. doi: 10.1186/1472-698X-13-23 (PMC3655915; doi:10.1186/1472-698X-13-23)
Supplement: Additional file 2 — COMMVAC CENTRAL search strategy. [file 1472-698X-13-23-S2.docx]

**SR-vacc**

| **ID** | **Search** | **Hits** |  |  |
| --- | --- | --- | --- | --- |
| #1 | [MeSH descriptor **Communication** explode all trees](http://onlinelibrary.wiley.com/o/cochrane/searchHistory?mode=runquery&qnum=1) | 7705 |  |  |
| #2 | [(health or patient or mediated or facilitated or augmentative or alternative or total or simultaneous or manual or mass or face-to-face or oral or mobile or cultural or risk or intervention or interaction or program* or skill or aid or tool or board or device or system or barrier) near/1 communication in Clinical Trials](http://onlinelibrary.wiley.com/o/cochrane/searchHistory?mode=runquery&qnum=2) | 1025 |  |  |
| #3 | [(*communicat* or messag* or *verbal* or written or writing or reading or language or speech or speak* or spoken or talk* or conversation or dialog* or discuss* or question* or voice or visual-perception or visual-art or gesture or symbol* or emblem or signage or metaphor* or feedback or listen* or negotiat* or notify* or notification or remind* or narrat* or music* or humor or humorous or cartoon or adverti* or persua* or interpreting or interpreters or interpret*-service or translat*-service or translating):kw,ti in Clinical Trials](http://onlinelibrary.wiley.com/o/cochrane/searchHistory?mode=runquery&qnum=3) | 27178 |  |  |
| #4 | [readability or intelligibility or credibility in Clinical Trials](http://onlinelibrary.wiley.com/o/cochrane/searchHistory?mode=runquery&qnum=4) | 332 |  |  |
| #5 | [(disclos* or *trust* or truth* or deceiv* or deception or misinform*):kw,ti in Clinical Trials](http://onlinelibrary.wiley.com/o/cochrane/searchHistory?mode=runquery&qnum=5) | 842 |  |  |
| #6 | [MeSH descriptor **Interpersonal Relations** explode all trees](http://onlinelibrary.wiley.com/o/cochrane/searchHistory?mode=runquery&qnum=6) | 3269 |  |  |
| #7 | [MeSH descriptor **Hospital-Patient Relations**, this term only](http://onlinelibrary.wiley.com/o/cochrane/searchHistory?mode=runquery&qnum=7) | 9 |  |  |
| #8 | [MeSH descriptor **Community-Institutional Relations**, this term only](http://onlinelibrary.wiley.com/o/cochrane/searchHistory?mode=runquery&qnum=8) | 152 |  |  |
| #9 | [(professional or physician or doctor or clinician or nurse or provider or practitioner or pediatrician or researcher) near/1 (patient or client or family or parent or subject) in Clinical Trials](http://onlinelibrary.wiley.com/o/cochrane/searchHistory?mode=runquery&qnum=9) | 3486 |  |  |
| #10 | [(consult* or referral or interview or history-taking or anamnesis):kw,ti in Clinical Trials](http://onlinelibrary.wiley.com/o/cochrane/searchHistory?mode=runquery&qnum=10) | 3932 |  |  |
| #11 | [(education* or teaching or learning or instruction*):kw,ti in Clinical Trials](http://onlinelibrary.wiley.com/o/cochrane/searchHistory?mode=runquery&qnum=11) | 22185 |  |  |
| #12 | [(health or patient or client) next (education or knowledge or promotion) in Clinical Trials](http://onlinelibrary.wiley.com/o/cochrane/searchHistory?mode=runquery&qnum=12) | 12824 |  |  |
| #13 | [(*education* or teaching or learning or instruction* or didactic or training or coaching or skills or online or web* or internet or video* or multimedia or multi-media or telephone) near/1 (intervention or session or course or program* or material or package or module or demonstration or method or process) in Clinical Trials](http://onlinelibrary.wiley.com/o/cochrane/searchHistory?mode=runquery&qnum=13) | 13801 |  |  |
| #14 | [((medical or continuing or residency or distance) near/2 education) or internship or inservice or in-service or staff-development or professional-development or mentor* or lifelong-learning in Clinical Trials](http://onlinelibrary.wiley.com/o/cochrane/searchHistory?mode=runquery&qnum=14) | 3939 |  |  |
| #15 | [(self next (teaching or education or instruction)) or autoinstruction* in Clinical Trials](http://onlinelibrary.wiley.com/o/cochrane/searchHistory?mode=runquery&qnum=15) | 124 |  |  |
| #16 | [((media or health) near/3 campaign) or (promotion next program*) or (community-based near/3 intervention) or (awareness near/3 (rais* or increas*)) in Clinical Trials](http://onlinelibrary.wiley.com/o/cochrane/searchHistory?mode=runquery&qnum=16) | 1005 |  |  |
| #17 | [marketing:kw,ti in Clinical Trials](http://onlinelibrary.wiley.com/o/cochrane/searchHistory?mode=runquery&qnum=17) | 304 |  |  |
| #18 | [(family or office or work* or school or faith or church or web or internet or phone or telephone or print or video) next based in Clinical Trials](http://onlinelibrary.wiley.com/o/cochrane/searchHistory?mode=runquery&qnum=18) | 2854 |  |  |
| #19 | [educational-status or literacy or critical-thinking in Clinical Trials](http://onlinelibrary.wiley.com/o/cochrane/searchHistory?mode=runquery&qnum=19) | 1221 |  |  |
| #20 | [(improv* or increas* or enhanc* or patient) near/3 (understanding or comprehension) in Clinical Trials](http://onlinelibrary.wiley.com/o/cochrane/searchHistory?mode=runquery&qnum=20) | 729 |  |  |
| #21 | [(information* next (intervention or service or center or system or dissemination or seeking or retrieval or transfer or campaign or provision or aid or material or sheet or pack* or letter)) in Clinical Trials](http://onlinelibrary.wiley.com/o/cochrane/searchHistory?mode=runquery&qnum=21) | 1025 |  |  |
| #22 | [(patient or client or health or medical or drug or preoperative or pre-operative or written or print* or online or on-line or visual* or auditory or provid* or present* or factual or objective) near/2 inform* in Clinical Trials](http://onlinelibrary.wiley.com/o/cochrane/searchHistory?mode=runquery&qnum=22) | 5731 |  |  |
| #23 | [*inform*:kw,ti in Clinical Trials](http://onlinelibrary.wiley.com/o/cochrane/searchHistory?mode=runquery&qnum=23) | 3274 |  |  |
| #24 | [((inform* or message or communicat* or effect or gain or positive or negative) near/2 fram*) or ((verbal or oral or written or text or information or data or dynamic or numerical or statistical or visual or graphic* or pictorial or audio* or video* or multimedia or multi-media or narrative) next (format or presentation or display)) in Clinical Trials](http://onlinelibrary.wiley.com/o/cochrane/searchHistory?mode=runquery&qnum=24) | 660 |  |  |
| #25 | [(checklist or prompt or ((drug or food or product) next label*)):kw,ti in Clinical Trials](http://onlinelibrary.wiley.com/o/cochrane/searchHistory?mode=runquery&qnum=25) | 374 |  |  |
| #26 | [(counsel* or advice or advis* or ((social or carer or caregiver or care-giver or patient) near/1 support*) or "supporting" or psychosocial or spiritual or ((social or pastoral) next care) or religion or chaplaincy or (behavior near/1 (modif* or chang*))):kw,ti in Clinical Trials](http://onlinelibrary.wiley.com/o/cochrane/searchHistory?mode=runquery&qnum=26) | 7274 |  |  |
| #27 | [(counsel*ing near/1 (session or method or patient or group or in-person)) or ((support or peer or self-help or self-care or self-management) near/2 (intervention or group or program* or project)) in Clinical Trials](http://onlinelibrary.wiley.com/o/cochrane/searchHistory?mode=runquery&qnum=27) | 4634 |  |  |
| #28 | [((social or community) near/2 network) or virtual-community or discussion-group in Clinical Trials](http://onlinelibrary.wiley.com/o/cochrane/searchHistory?mode=runquery&qnum=28) | 360 |  |  |
| #29 | [(self-care or self-management):kw,ti in Clinical Trials](http://onlinelibrary.wiley.com/o/cochrane/searchHistory?mode=runquery&qnum=29) | 2263 |  |  |
| #30 | [(motivat* or incentive or goal):kw,ti in Clinical Trials](http://onlinelibrary.wiley.com/o/cochrane/searchHistory?mode=runquery&qnum=30) | 3645 |  |  |
| #31 | [MeSH descriptor **Communications Media** explode all trees](http://onlinelibrary.wiley.com/o/cochrane/searchHistory?mode=runquery&qnum=31) | 5318 |  |  |
| #32 | [(mass or communication or electronic or digital or multi or print* or social or new) next media in Clinical Trials](http://onlinelibrary.wiley.com/o/cochrane/searchHistory?mode=runquery&qnum=32) | 342 |  |  |
| #33 | [((print* next (material or message or feedback or based)) or paper-based or postal or mail* or letter or correspondence or (paper near/2 pen*) or publication or newsletter or brochure or booklet or pamphlet or leaflet or flyer or handout or poster or billboard* or illustrat* or picture or pictogram* or graphic* or icon*):kw,ti in Clinical Trials](http://onlinelibrary.wiley.com/o/cochrane/searchHistory?mode=runquery&qnum=33) | 3768 |  |  |
| #34 | [(radio or television or audio* or video* or tape or recording or cassette or cd-rom or dvd or motion-picture or film or multimedia or hypermedia or game or telephon* or phone or sms or i-pod or ipod or mp3-player or hotline or answering-service or internet or web* or online or on-line or electronic or blog* or bulletin-board or telemedicine or telehealth or telecare or telemanag* or virtual):kw,ti in Clinical Trials](http://onlinelibrary.wiley.com/o/cochrane/searchHistory?mode=runquery&qnum=34) | 11317 |  |  |
| #35 | [(e next (mail or prescrib* or health)):kw,ti in Clinical Trials](http://onlinelibrary.wiley.com/o/cochrane/searchHistory?mode=runquery&qnum=35) | 77 |  |  |
| #36 | [MeSH descriptor **Computer Systems** explode all trees](http://onlinelibrary.wiley.com/o/cochrane/searchHistory?mode=runquery&qnum=36) | 2081 |  |  |
| #37 | [MeSH descriptor **Software**, this term only](http://onlinelibrary.wiley.com/o/cochrane/searchHistory?mode=runquery&qnum=37) | 629 |  |  |
| #38 | [(computer* near/1 (system or network or program* or terminal or interface or interact* or handheld or intervention or strategy or therapy or graphic or simulation or searching or mediated or based or generated or tailored or communication or assisted-instruction)):kw,ti in Clinical Trials](http://onlinelibrary.wiley.com/o/cochrane/searchHistory?mode=runquery&qnum=38) | 3402 |  |  |
| #39 | [touch-screen or digital-assistant or pda or blackberry or mobile-device or laptop or notebook in Clinical Trials](http://onlinelibrary.wiley.com/o/cochrane/searchHistory?mode=runquery&qnum=39) | 403 |  |  |
| #40 | [((automat* or interactive*) near/3 (telephon* or phone or voice or hotline or hot-line)) or ((voice or speech) next (response or recognition or messag* or system or technolog*)) in Clinical Trials](http://onlinelibrary.wiley.com/o/cochrane/searchHistory?mode=runquery&qnum=40) | 283 |  |  |
| #41 | [(participation or advocacy or consumer or empower*):kw,ti or ((increas* or promot* or support*) near/2 participation) in Clinical Trials](http://onlinelibrary.wiley.com/o/cochrane/searchHistory?mode=runquery&qnum=41) | 2398 |  |  |
| #42 | [MeSH descriptor **Informed Consent** explode all trees](http://onlinelibrary.wiley.com/o/cochrane/searchHistory?mode=runquery&qnum=42) | 445 |  |  |
| #43 | [(informed next (consent or choice or decision)):kw,ti in Clinical Trials](http://onlinelibrary.wiley.com/o/cochrane/searchHistory?mode=runquery&qnum=43) | 673 |  |  |
| #44 | [(patient or person or family or client) next (cent*red or focus*ed or oriented) in Clinical Trials](http://onlinelibrary.wiley.com/o/cochrane/searchHistory?mode=runquery&qnum=44) | 733 |  |  |
| #45 | [therapeutic next (relation* or alliance or partner*) in Clinical Trials](http://onlinelibrary.wiley.com/o/cochrane/searchHistory?mode=runquery&qnum=45) | 215 |  |  |
| #46 | [MeSH descriptor **Decision Making** explode all trees](http://onlinelibrary.wiley.com/o/cochrane/searchHistory?mode=runquery&qnum=46) | 1971 |  |  |
| #47 | [decision next (making or support or aid or tool or technology) in Clinical Trials](http://onlinelibrary.wiley.com/o/cochrane/searchHistory?mode=runquery&qnum=47) | 2685 |  |  |
| #48 | [(shared or joint or informed) near/3 decision in Clinical Trials](http://onlinelibrary.wiley.com/o/cochrane/searchHistory?mode=runquery&qnum=48) | 293 |  |  |
| #49 | [cultural* next (competen* or sensitiv* or appropriate*) in Clinical Trials](http://onlinelibrary.wiley.com/o/cochrane/searchHistory?mode=runquery&qnum=49) | 242 |  |  |
| #50 | [(cultural* or linguistic* or language) near/3 (service or care or intervention or message) in Clinical Trials](http://onlinelibrary.wiley.com/o/cochrane/searchHistory?mode=runquery&qnum=50) | 280 |  |  |
| #51 | [(#1 OR #2 OR #3 OR #4 OR #5 OR #6 OR #7 OR #8 OR #9 OR #10 OR #11 OR #12 OR #13 OR #14 OR #15 OR #16 OR #17 OR #18 OR #19 OR #20 OR #21 OR #22 OR #23 OR #24 OR #25 OR #26 OR #27 OR #28 OR #29 OR #30 OR #31 OR #32 OR #33 OR #34 OR #35 OR #36 OR #37 OR #38 OR #39 OR #40 OR #41 OR #42 OR #43 OR #44 OR #45 OR #46 OR #47 OR #48 OR #49 OR #50)](http://onlinelibrary.wiley.com/o/cochrane/searchHistory?mode=runquery&qnum=51) | 114887 |  |  |
| #52 | [MeSH descriptor **Immunization** explode all trees](http://onlinelibrary.wiley.com/o/cochrane/searchHistory?mode=runquery&qnum=52) | 3538 |  |  |
| #53 | [MeSH descriptor **Vaccines** explode all trees](http://onlinelibrary.wiley.com/o/cochrane/searchHistory?mode=runquery&qnum=53) | 6434 |  |  |
| #54 | [immuniz* or immunis* or immunotherap* or vaccin* or inoculat*](http://onlinelibrary.wiley.com/o/cochrane/searchHistory?mode=runquery&qnum=54) | 14324 |  |  |
| #55 | [(#52 OR #53 OR #54)](http://onlinelibrary.wiley.com/o/cochrane/searchHistory?mode=runquery&qnum=55) | 14344 |  |  |
| #56 | [(#51 AND #55)](http://onlinelibrary.wiley.com/o/cochrane/searchHistory?mode=runquery&qnum=56) | 814 |  |  |
