# Supplementary material for: “Communicate to vaccinate”: the development of a taxonomy of communication interventions to improve routine childhood vaccination
Source: BMC Int Health Hum Rights. 2013 May 11;13:23. doi: 10.1186/1472-698X-13-23 (PMC3655915; doi:10.1186/1472-698X-13-23)
Supplement: Additional file 3 — COMMVAC data extraction form. [file 1472-698X-13-23-S3.doc]

| **Sample** | |
| --- | --- |
| Full citation |  |
|  |  |
| Name of person extracting data |  |
| Date form completed |  |
| Notes |  |
|  |  |
| **Study** |  |
|  |  |
| Type of study (as described) |  |
| Study aim (as described) |  |
| Study duration |  |
| Vaccine type and dose |  |
| Vaccine delivery strategy |  |
| Age of individuals receiving vaccine |  |
| Notes |  |
|  |  |
| **Participants** |  |
|  |  |
| Population targeted |  |
| Inclusion criteria |  |
| Exclusion criteria |  |
| Country or region |  |
| Low-income country? |  |
| Middle-income country? |  |
| Geographic description |  |
| Race/ethnicity/religion |  |
| Level of education of recipients |  |
| Were any populations specifically excluded from the study |  |
| Notes |  |
|  |  |
| **Intervention OVERVIEW** |  |
|  |  |
| Description of interventions |  |
| Problem intervention is attempting to address |  |
| Aim of intervention: Implied or stated purpose |  |
| Outcomes (as described) |  |

| **Intervention #1** |  |
| --- | --- |
|  |  |
| Description of intervention |  |
| Problem intervention is attempting to address |  |
| Aim of intervention: Implied or stated purpose |  |
| Setting of intervention |  |
| Format |  |
| Providers / deliverers |  |
| Timing & frequency of delivery |  |
| Content of communication |  |
| Resource requirements for delivery of the intervention |  |
| Duration of follow-up |  |
| Details of control/usual or routine care |  |
| Co-interventions (as described) |  |
| Outcomes (if unique to this intervention) |  |
| Consumer involvement |  |
| Underlying theoretical or conceptual model |  |
| Categorisation of intervention |  |
| Parties involved & direction of communication |  |
| Notes |  |

| **Potential Intervention #1** |  |
| --- | --- |
|  |  |
| Description of intervention |  |
| Problem intervention is attempting to address |  |
| Aim of intervention: Implied or stated purpose |  |
| Setting of intervention |  |
| Format |  |
| Providers / deliverers |  |
| Timing & frequency of delivery |  |
| Content of communication |  |
| Resource requirements for delivery of the intervention |  |
| Duration of follow-up |  |
| Details of control/usual or routine care |  |
| Co-interventions (as described) |  |
| Outcomes (if unique to this intervention) |  |
| Consumer involvement |  |
| Underlying theoretical or conceptual model |  |
| Categorisation of intervention |  |
| Parties involved & direction of communication |  |
| Notes |  |
