# Supplementary material for: “Communicate to vaccinate”: the development of a taxonomy of communication interventions to improve routine childhood vaccination
Source: BMC Int Health Hum Rights. 2013 May 11;13:23. doi: 10.1186/1472-698X-13-23 (PMC3655915; doi:10.1186/1472-698X-13-23)
Supplement: Additional file 4 — COMMVAC deliberative forum reports. [file 1472-698X-13-23-S4.pdf]

COMMVAC deliberative forum report

New York, 8th of June 2011

I. Process

Participants were invited about two weeks in advance. While we had initially compiled a tentative list of approximately 15 people, 6 of them (as well as the International Union for Health Promotion and Education (IUHPE) facilitator) were able to attend the meeting.

Participants included both members of the IUHPE Board of Trustees and representatives from UNICEF[1] (see list below).

Participants received material on the project 10 days prior to the meeting, and they were presented with the specific questions that would addressed during the dialogue with them including a discussion on the taxonomy, the identification of priorities for systematic reviews as well as priorities for further primary research.

The material received included: a one page briefing on the project, an agenda, as well as the research summary (presenting the taxonomy development and the evidence base mapping).

We began the meeting by providing participants some further background to the project (with power point presentation) and emphasizing how these stakeholders' consultations constitute a critical step for the project in order to guarantee that the reviews are produced in a way that relevant evidence can be useful for different stakeholders and easily picked up for the purposes of implementation by immunization programme planners, managers and policy makers.

The following questions were then addressed with participants:

- Does the taxonomy capture the key intervention groups? Are there important interventions groups missing from the taxonomy?
- What are the overall priority intervention groups relevant to low and middle income countries (LMICs) for systematic reviews of effects? How can this help us to evaluate priorities
- What are the priorities for systematic reviews of effects?
- What are the priority intervention groups for further primary research?

Participants engaged very actively in the discussion, there was clearly a lot of interest and issues to be raised. Given the fact that the questions were all interrelated, the discussion did not follow any specific structure. The dialogue was open so as to allow participants to freely express their views, experiences and recommendations.

---

[1] See annex I.

At the end of the discussion we provided participants with color cards and asked them, in light of the general discussion, to write down their personal input to the three questions (i.e. priorities intervention groups for LMICs, priorities for SR of effects, priorities for further primary research).

The following report presents the main input and points that arose from the general discussion as well as the input provided by the written cards.

II) Discussion around the taxonomy and with respect to the intervention groups that are relevant to LMICs

a. Categorization and framing of the taxonomy

- The taxonomy includes the main categories but it could be nuanced a bit further and made more specific in some places.
- It might be useful to look at barriers and problems as a way to ensure that the evidence base summaries respond to these. This would also contribute to encourage decision makers to identify what problem they face in particular and to look for what evidence lies in this particular area (taking into account both contextual elements and potential mix of solution/strategies).
- It was acknowledged that systematic reviews traditionally define the intervention and the intervention effect of interest for the review. Systematic reviews rarely ask the question concerning whether an intervention is responding to a problem or the driver of a problem. It was therefore suggested to reframe the labels, aligning the taxonomy along a problem-oriented nature to reflect that the *purpose* issues include a more explicit expression of the assumptions behind them. The names of these categories in the taxonomy could be revised to reflect the nature of the problem to which an intervention that could be found in that category would respond to, how it would target the problem.

b. Further clarification/specification/distinction in the taxonomy

- There is **inconsistency in the taxonomy** as some categories are stated more as objectives (i.e. to increase access, to minimise risks or harms) without a sense of action, while others focus more on action (i.e. to inform or educate would imply to increase knowledge); while “to support” could actually be related to several different objectives.
- Further clarification about the **distinction between *providers* and *consumers*** would be useful. At present the taxonomy does not sufficiently or explicitly grasp providers perspectives in terms of communication, education and behavior change (communication to providers, how providers are trained and prepared to address rumors, side effects, reminders etc) and in terms of incentives (which are relevant both for *recipients* AND *consumers*). Moreover the category “To teach skills” mixes providers and consumers – it might be better to separate them more clearly.
- The distinction between **the supply and demand sides** needs to be made (as the current taxonomy does not explicitly capture the dynamic relationship between the two).
    - For example: What are the dynamics of provider and health worker motivation in terms of vaccination uptake? The experience and provision of care as well as the communication issue influence the demand side.

- Some interventions are interventions concerned with organising of supply side, and not related to communication (i.e. most of the ones under to increase access) – these may not be necessary for this review.
- If the supply side organisation interventions were going to be included, then
- It would be useful to provide a better separation of demand side and supply side – in fact, some of the current categories might fit better under 'to support' (e.g. transportation assistance, childcare services).
- health workers motivation should get attention.

c. Suggestion of new categories for the taxonomy

Participants suggested adding the following categories which are particularly relevant in the context of LMICs

- *To change social norms* (attitudes, values – social and cultural norms)
  - Within institutions such as the Ministry of Health
  - Within communities
- *To change attitudes*
- *To build trust*
  - Reassurance
  - Improve quality
- '*To minimise risks or harms'* which could be linked to 'to build trust'

d. **Priority intervention groups relevant to LMICs**

The following priority intervention groups/categories were suggested:

- To support
- Existing enabling environment to support vaccination
- To involve the community – which will help to develop /build trust—(which might then become another taxonomy)
- To involve the community in research (and area we need more evidence on).
- To teach skills (and change attitudes)
- To minimize risk of harms transitions/"to build trust"
- Health/service provider skills, attitudes and availability
- To improve quality
- To encourage continuity
- To increase access to adds
- Common Media campaign and use in community structures
- Web based approaches are not common but there is for example evidence emerging on the use of SMS (as reminders for vaccination)
- Power relationship between husband and wife

III) Priorities for Systematic Reviews of effects

A) Suggested priorities for the systematic reviews

- The use of new technologies such as cell phones for example[2]

[2] Note that a text messaging programme in Kenya and Uganda is being documented by UNICEF and others

- "Mix" of communication channels –what's the optimal mix, cost effectiveness of using different and mixed approaches, interventions and synergies[3]
- The role of community health workers as interface
- Immunization days as a rallying point for re energizing commitment around vaccination (approach widely developed in LMICs- to what extent is it really effective?)
- Usefulness of mass campaigns –an approach that is extensively used but what is its real impact?
- School based interventions – school as a platform/setting for communication interventions for vaccination.
- Role of supporting interventions, networks, mechanisms, dynamics (taking into account the role of religious/local traditional communication networks)
- The role of policy support to immunization
- The importance of political will and support at all levels
- Investigation on the reasons for dropout rates + response as a critical issue for LMICs
- Service and resources availability
- The SR could take specific vaccines focus/have subcategories with:
    - Measles (evidence from MDG country reports and UN reports)
    - Polio (based on information provided by UNICEF)
    - DPT (high coverage)

B) Comments and issues to be taken into consideration

- The critical need and importance of documenting community based interventions (communication for social change) related to immunization – this relates to the discussion held in Melbourne on other ways of pulling evidence that should be considered in the realm of the project or as a next and complementary step to the project. Note that the IUHPE has developed a tool for documenting health promotion case studies in the Eastern Mediterranean region that could support this process.
- Try to specify the contextual elements that contribute to the effectiveness of interventions (cultural, institutional systems, political and economic environment) as well as the supporting conditions (such as legislation for example).
- Examine the gender dimensions of all studies.
- Where should a communication interventions start? Should it be through antenatal or postnatal care. The importance of prenatal care in that respect is not sufficiently acknowledged.
- It might be interesting to have a look at the indicators coming from the monitoring and evaluation of measles in the context of MDGs as countries engaged in the MDGs are reporting annually on these (this would constitute potential source of readily available data).

[3] UNICEF is currently developing a paper that describes the different channels of communication and mixed approaches.

C) Discussion related to effect

What is the effect that we are looking at in the context of this research and what does uptake refer to in this regard? Are we referring to increased coverage, improved reminder rates?

The links between the research review priorities and priorities for intervention groups might require revising the projects objective/expected outcomes accordingly. Is the research going to lead to increase vaccination uptake?

IV) Priorities for further primary research

Participants expressed that the following would be areas of interest for further primary research as some of these issues are critical and not appropriately documented and evaluated.

- The importance of **cultural beliefs and practices/social norms** that prevail in a community –both in terms of barriers and opportunities- need to be further explored. Communication interventions that focus on knowledge and information are not sufficient. There needs to be more attention paid to attitudes and values.
- The **involvement of community**– how it was done? At what stage, who (such as a women with disability for example).
- Further explore the **role of different stakeholders** such as donors, NGOs, CBOs ,faith based organization in terms of support, design, management, implementation of communication strategies.
- The **equity issue** in the eradication of specific disease (such as polio for example) where one can see a social gradient as it is the case in other health outcomes.
- **Positive deviance**: Explore if there are cases where immunization rate stayed high during difficult times (such as rumors, internal political conflict etc). This could constitute an interesting base for further research to find out what communication activities were done to sustain/enable routine immunization).
- What is the optimist **mix of communication strategies** and interventions
- Interface between **supply and demand** side (communications, interaction)
- Explore the source, factor, context for motivated **human resources/providers** of immunization programmes
- How **public trust** is built

V. Evaluation on how the dialogue was conducted

The material sent in advance was very useful in terms of preparation, and it was clearly presented.

The power point presentation provided a good foundation and start to the meeting. It enabled clarification of the project's background, how and where the materials were developed so far, and how the consultation forum would fit in the overall project.

Participants appreciated the open nature of the dialogue, as it enabled them to address key issues, and draw upon the full range of people's experience and expertise.

They also appreciated the opportunity to be informed about this project, and the possibility of linking it to other initiatives or grey literature and as a critical input for the field of immunization programs. The project was perceived as providing some new and innovative approach to these issues and they were impressed by the quality of the work undertaken so far.

Participants also appreciated the transparency on the barriers/limitations and challenges that the project encounters. It was useful to acknowledge these in the discussion and in thinking of future opportunities.

VI. Follow up with participants

There was a strong interest from participants to be kept informed about the project progress, to be consulted again at critical times both for input on specific outputs (such as the evidence summaries for example), to provide contacts of individuals that could participate in the online forum, to participate themselves in the online discussion if useful and to make linkages with other interesting initiatives.

Participants also mentioned some initiatives and documents that could be useful to the project including:

- The UNICEF “communication framework for new vaccinations”
- The report on the text messaging projects (Uganda and Kenya) that should soon be available
- Annual reporting from African countries and others on progress on MDG 4 with regard to measles specifically
- UNICEF country good practice from child and maternal health programmes
- John Hopkins University and other partners are currently developing a research initiative on immunization where some important links could be made
- Review from OB – we need to double check what this was referring to.

COMMVAC Deliberative Forum, New York 8th of June 2011, Hotel Lucerne

List of Participants

Amuyunzu-Nyamongo, Mary
Executive Director, African Institute for Health and Development (AIHD), Kenya
Member of the IUHPE Board of Trustees
Email: manyamongo@yahoo.com

Bensaude De Castro Freire, Sara
Scientific projects and publications Coordinator, IUHPE HQ, France
Email: sbensaude@iuhpe.org

Claycom, Paula
Senior Advisor, Communication for Development, UNICEF HQ, USA
Email: pclaycomb@unicef.org

Jones, Catherine
Programme Director, IUHPE HQ, France
Email: cjones@iuhpe.org

Kagwa, Paul
Assistant Commissioner, Health Promotion and Education, Ministry of Health, Uganda
Member of the IUHPE Board of Trustees
Email: paulkagwa@yahoo.co.uk

Lin, Vivian
Professor of Public Health, School of Public Health, La Trobe University, Australia
IUHPE Global Vice- President for Scientific Affairs
Member of the IUHPE Board of Trustees
Email: v.lin@latrobe.edu.au

McKay, Susan
Communication for Vaccination specialist, UNICEF HQ, USA
Email: smackay@unicef.org

Manoncourt, Erma
Management Development Specialist / Associate- Development Works International
IUHPE Global Vice President for Communications
IUHPE Chair of the Global Working Group on Social Determinants of Health
Member of the IUHPE Board of Trustees
Email: emanoncourt@yahoo.com

Sakaedani- Petrovic, Akiko
Communication for Develpment specialist, UNICEF HQ, USA
Email: asakaedani@unicef.org

COMMVAC deliberative forum report

Ottawa, 28th of June 2011

I. *Process*

Participants were invited to the meeting about ten days in advance. We selected participants attending the Francophone International Conference on Local and Regional Health Programmes (http://www.plrs-ottawa2011.com/) working in (or on) sub-Saharan Africa.

While we had initially compiled a tentative list of approximately 15 people, 10 of them as well as the International Union for Health Promotion and Education (IUHPE) facilitator) were able to attend the meeting.

Participants mostly included health programme mangers working either at the regional level (such as in provincial health services) or at the national level (at the Ministry of Health) and all coming from sub-Saharan Africa (see list below).

Participants received material on the project six days prior to the meeting, and they were presented with the specific questions that would be addressed during the dialogue including a discussion on the taxonomy, the identification of priorities for systematic reviews as well as priorities for further primary research.

The material received (in French) included: a one page briefing on the project, an agenda, as well as the research summary (presenting the taxonomy development and the evidence base mapping).

The meeting was conducted in a very similar way than consultation we had in New York. The meeting took place in the evening (after an intense day at the conference) and was therefore scheduled for an hour and fifteen minutes.

We began the meeting by providing participants some further background to the project (with power point presentation) and emphasizing how these stakeholders' consultations constitute a critical step for the project in order to guarantee that the reviews are produced in a way that relevant evidence can be useful for different stakeholders and easily picked up for the purposes of implementation by immunization programme planners, managers and policy makers.

The following questions were then addressed with participants:

- Does the taxonomy capture the key intervention groups? Are there important interventions groups missing from the taxonomy?
- What are the overall priority intervention groups relevant to low and middle income countries (LMICs) for systematic reviews of effects? How can this help us to evaluate priorities
- What are the priorities for systematic reviews of effects?
- What are the priority intervention groups for further primary research?

Participants engaged very actively in the discussion, there was clearly a lot of interest and issues to be raised as these issues were recognized to be critical for the African context. Given the fact that the questions were all interrelated, the discussion did not follow any specific structure. The dialogue was open so as to allow participants to freely express their views, experiences and recommendations.

The content of the discussion slightly differed from the one we had in New York. This is most probably due to the profile of the participants who were not experts in the field of communication or vaccination but rather health programme managers and coordinators. There was less focus on the taxonomy as such and more discussion around the priority intervention groups and priorities for the SR of effects. While the discussion differed it also converged in many ways, as illustrated in the report, and a lot of the issues and concerns addressed were similar to the ones discussed in New York.

At the end of the discussion we provided participants with color cards and asked them, in light of the general discussion, to write down their personal input to the three questions (i.e. priorities intervention groups for LMICs, priorities for systematic reviews of effects, priorities for further primary research).

The following report presents the main input and points that arose from the general discussion as well as those provided by the written cards.

II) Discussion around the taxonomy and with respect to the intervention groups that are relevant to LMICs

a. General comments regarding the taxonomy

- The taxonomy is long and complex
- Not all intervention groups as captured in the taxonomy seem relevant to LMICs context and particularly to sub-Saharan Africa (such as the use of internet for example).
- Greater attention could be paid to issues related to advocacy towards community and religious leaders who have a great influence to play in immunization programs. This links to the creation of social norms (such as traditional leaders, communities, religious leaders) and community mobilization (which is essential in terms of sustainability).
- Non verbal communication could more explicitly be included
- In the African context, it would seem essential to better articulate different types of communications targeted at the population by developing a strategy of communication through development in a integrated fashion (there tends to be an overload of communications).
- It is an issue when immunization is considered through different programs and communication strategies as an individual issue and choice (i.e. the mother chooses to bring or not her child to immunization). It is in reality much more complex and dependent on structural factors, such as the availability of transport, the cultural acceptability of services, the vision of other influential members of the community

on immunization programs (such as grandmothers, husbands for example). This is somehow not sufficiently explicit in the taxonomy.

b. Further clarification/specification/distinction in the taxonomy

- Participants suggested further clarification about the **distinction between *providers* and *consumers*** would be useful. At present the taxonomy does not sufficiently or explicitly grasp providers perspectives in terms of communication, education and behavior change (communication to providers, how providers are trained and prepared to address rumors, side effects, reminders etc, values and attitudes of providers). An essential component of immunization communication programs should address provider's perspective and encourage them to better communicate. Community agents are an essential part of immunization programs. The perception of service providers by the community is also very important. Who are the service providers? Are they members of the community or external people? All these elements have a great influence on immunization programs perception by the community and should be taken into consideration when developing communication strategies.
- The distinction between **the supply and demand sides** needs to be made (as the current taxonomy does not explicitly capture the dynamic relationship between the two).
    - For example: What are the dynamics of provider and health worker motivation in terms of vaccination uptake? The experience and provision of care as well as the communication issue influence the demand side

It is essential to link promotion and communication strategies to issues related to the supply of immunization. In that sense they should be considered in a holistic fashion.

c. Suggestion of new categories for the taxonomy

- The creation of social norms (such as traditional leaders, communities, religious leaders)

d. Priority intervention groups relevant to LMICs

The following priority intervention groups/categories were suggested:

- Community mobilization/network/involvement (essential in terms of sustainability) including the role of religious leaders, speaking communities, women's self help group, parents etc)
- Grandmothers, mothers are essential and influential actors when it comes to health, immunization and misconception about these. It is therefore essential to target them and reach them in settings where they are most confident.
- TV mass campaigns
- The use of modern technology seems particularly relevant and offers a great potential in the African context. In that perspective some efforts to approach mobile operators could be considered. It is nevertheless important to acknowledge that such strategies would only reach certain categories of the population and should therefore be considered in conjunction with other strategies.
- To increase access. Communication strategies targeted/addressing remote areas which are often neglected

- To minimize risk
- To recall/remind
- To teach skills (this strategy and approach is not widely used)
- To support (this strategy and approach is not widely used)
- Strategies that would address the wider and structural context in which people live (economic constraints and all resulting issues) although these might not directly link to communications issues.

III) Priorities for Systematic Reviews of effects

A) Suggested priorities for the systematic reviews

-To educate and raise awareness on the use of vaccines and related misconceptions about secondary effects
- The use of modern technologies
- Communication targeted at service providers
- To minimize risks or damage
- Look at issues of isolation and the effects of efforts to invest in mobile clinic strategies (and other similar initiatives) to facilitate access to the programs for isolated population.

B) Discussion related to effect

- As in the New York forum, participants noted that the links between the research review and priorities for intervention groups might require revising the projects objective/expected outcomes accordingly. Is the research going to lead to increase vaccination uptake? The principal research question might need to be reconsidered.
- Why does the project only target children and does not take into consideration women (immunization of women is also an essential strategy to decrease mortality in LMICs)
- In the process of conducting the systematic reviews, it would be essential to refer to empirical and qualitative data and identity strategies that would enable to collect and grasp knowledge that is not documented through more 'formal' and traditional means of evaluation such as trials. There are a substantial number of activity reports and other types of documentation related to experiences and evaluation of communication strategies and immunization programs but these are not necessarily included in these types of research. This issue was also emphasized in NY and briefly discussed in Melbourne and could be picked up during the next partners meeting in Madrid.

IV) Priorities for further primary research

Participants expressed that the following would be areas of interest for further primary research as some of these issues are critical and not appropriately documented and evaluated.

- The **involvement of community**– how it was done? At what stage, who? (mothers, grandmothers, religious leaders etc).

- Take into account **perspective of users**, the experience of individuals, family and community with respect to immunization programs, health services, communication strategies etc.
- Further explore the involvement of **different types of stakeholders** (including modern and traditional stakeholders) such as donors, NGOs, CBOs ,faith based organization in terms of support, design, management, implementation of communication strategies.
- The importance of developing **research at the local level** related to local interventions
- To explore **innovation strategies** for funding immunization programs
- It would be interesting to further develop **action research**

V. Evaluation on how the dialogue was conducted

The material sent in advance was very useful in terms of preparation, and it was clearly presented. Participants appreciated the open nature of the dialogue, as it enabled them to address key issues, and draw upon the full range of people's experience and expertise.

They also appreciated the opportunity to be informed about this project, and the possibility of linking it to other initiatives or grey literature and as a critical input for the field of immunization programs. The project was perceived as providing some new and innovative approach to these issues and they were impressed by the quality of the work undertaken so far.

Efforts, such as these consultation, seem essential in order to collect and take into consideration the perspectives and issues encountered by stakeholders. Participants encouraged the approach taken and called for similar approaches at different stages of the project. They expressed a lot of interest to be informed on the project developments and engaged in other consultations as appropriate.

VI. Follow up with participants

There was a strong interest from participants to be kept informed about the project progress, to be consulted again at critical times both for input on specific outputs (such as the evidence summaries for example), to participate in the online forum and make linkages with other interesting initiatives.

Participants also mentioned some initiatives and documents that could be useful to the project including:

- The Health Observatories in Africa
- An Organisation supported by the Fondation Helwet in Ghana: the **Union for African Population Studies**. (The Union for African Population Studies (UAPS) is a pan-African, non-profit, scientific organization whose purpose is to promote the scientific study of population in Africa. For nearly 25 years, UAPS has fostered the networking of researchers, policy makers, and other stakeholders across geographic and language barriers in order to improve research capacity and promote evidence-based policies and programs in population and development in Africa. The UAPS network of nearly 1200 members is able to link multidisciplinary scholars and other professionals for knowledge sharing and to facilitate collaborative research opportunities.http://uaps-uepa.org)
- National monitoring systems

- The Center of information and communication in health (Mali), which is a recent structure that focuses among other things on immunization: http://www.sante.gov.ml/index.php?option=com_content&task=view&id=72&Itemid=74

COMMVAC Deliberative Forum, Ottawa, 28 June 2011, Hôtel Hilton Lac Leamy

Liste des participants

Athie, Cheikh Tidiane
ONG ACDEV Action et Développement
Sénégal
Email : acdev@orange.sn

Bah, Aissatou Tinka
Centre de Coopération Internationale en Santé et Développement (CCISD)
Canada
Email : aissatou-tinka.bah@ccisd.org

Bensaude De Castro Freire, Sara
Coordinatrice des projets et publications scientifiques, UIPES
France
Email: sbensaude@iuhpe.org

Dedewanou, Gilda Raoul J.S
Ministère de la santé
Bénin
Email : degildas@yahoo.fr

Abdou Salam Fall
Sociologue
Sénégal
asfall@refer.sn

Housna, Assomption
Ministère de la santé
Bénin
Email: ass_hounsa@yahoo.fr

Jones, Catherine
Directrice des programmes, UIPES
France
Email: cjones@iuhpe.org

Maiga, Abdoulaye
Direction régionale du développement social et de l'économie solidaire
Mali

Sidibé, Mody
Médecin responsable du VIH/SIDA
Direction Régionale de la Santé de Kayes
Mali
Email : modysi@yahoo.fr

Yatara, Moussa
Directeur de la Direction Régionale de la Santé
Mali
